# Changes in symptoms of asthma and rhinitis by sensitization status over ten years in a cohort of young Chilean adults

**DOI:** 10.1186/s12890-016-0273-6

**Published:** 2016-08-08

**Authors:** Vanessa Garcia-Larsen, James F. Potts, Stefano Del Giacco, Patricia Bustos, Patricia V. Diaz, Hugo Amigo, Manuel Oyarzun, Roberto J. Rona

**Affiliations:** 1 Respiratory Epidemiology, Occupational Medicine, and Public Health Group, National Heart & Lung Institute, Imperial College London, Emmanuel Kaye Building, Manresa Road, London, SW3 6LR UK; 2 Department of Medical Sciences “M Aresu”, University of Cagliari, Asse Didattico “E1” – Cittadella Universitaria, Monserrato, Cagliari 09042 Italy; 3 Department of Nutrition, Faculty of Medicine, University of Chile, Independencia 1027, Santiago, Chile; 4 Institute of Bio-Medical Science, Faculty of Medicine, University of Chile, Independencia 1027, Santiago, Chile; 5 Department of Psychological Medicine, Weston Education Centre, King’s College London, Cutcombe Road, London, SE5 9RJ UK

**Keywords:** Allergy, Asthma symptoms, Change, Cohort, Young adults, South America

## Abstract

**Background:**

We investigated the net changes in prevalence of symptoms of asthma and rhinitis over 10 years in a cohort of young by baseline sensitization status.

**Methods:**

One thousand one hundred ninety three Chilean adults subjects aged 22–28 living in a semi-rural area of central Chile answered a lifestyle and the European Community Respiratory Health Survey (ECRHS) questionnaires. Bronchial hyper-responsiveness (BHR) and skin prick test (SPT) to eight allergens were measured at baseline in 2001. Ten years later, 772 participants completed the questionnaires again. Estimates of adjusted net changes in prevalence of symptoms by sensitization status at baseline and association between sensitization status at baseline and respiratory symptoms ten years later were assessed.

**Results:**

A quarter of the participants were sensitized to at least one allergen in 2001. Prevalence of wheeze had a net change per year of −0.37 % (95 % Confidence Interval −0.71 to 0.02 %; *p* = 0.067). Self-reported nasal allergies in the last 12 months increased by 0.83 % per year (95 % CI 0.49 to 1.17 %; *p* < 0.001). Those sensitized to either cat fur (OR 1.76; CI 1.01 to 3.05), cockroach, (OR 2.09; 1.13 to 3.86) blend of grass and pollens (1.78; 95 % CI 1.08 to 2.92), or weeds (OR 1.77; 95 % CI 1.01 to 3.12) in 2001 were more likely to have wheeze in the last 12 months 10 years later.

**Conclusion:**

Symptoms of asthma remained stable or slightly changed over 10 years in adults, whilst rhinitis and nasal allergies greatly increased. Being sensitized to at least one allergen is a risk factor for persistent symptoms of asthma and rhinitis, but not for determining net changes of symptoms over time. The underlying causes for the contrasting trends between asthma and nasal allergy are unknown.

**Electronic supplementary material:**

The online version of this article (doi:10.1186/s12890-016-0273-6) contains supplementary material, which is available to authorized users.

## Background

Countries with emerging and fast growing economies have similar prevalence of asthma to developed countries [1, 2]. In Chile, the prevalence of asthma in children is 22 % [1] whilst in adults it ranges between 16 and 28 % depending on the epidemiological approaches used to measure it [3, 4].

Longitudinal studies on changes of asthma symptoms, nasal allergy and sensitization are scant, with most of the evidence coming from European countries. The follow-up analysis of the European Community Respiratory Health Survey (ECRHS) cohort showed that adults aged 25–49 were more likely to have symptoms of asthma at 10 year follow-up if they were sensitized to pets at baseline [5]. Similarly, another longitudinal study in Italian adults showed that sensitization to indoor allergens was associated with having a higher prevalence of symptoms ten years later [6]. A higher prevalence of respiratory symptoms was also observed at follow-up in other two studies with selected samples from existing cohort studies [7, 8]. The ECRHS has also shown that symptoms of asthma have remained stable over time in middle aged adults [9]. However, the ECRHS results, the main body of evidence, need to be replicated to know the extent to which they conform a general pattern.

The Limache (Chile) Cohort study started in 2001 with the aim of identifying early life and current risk factors for asthma in young adults from a semi-rural area [10]. In 2011, we examined this cohort again to study respiratory symptoms using the ECRHS questionnaire [11]. This follow-up study allows an assessment of the evolution of symptoms of the disease over time. The aims of this study were to investigate the net changes in prevalence of respiratory symptoms and nasal allergies within a period of ten years in a cohort of young adults, according to sensitization status based on skin prick test (SPT) to eight allergens. We also aimed to assess whether the sensitization status in 2001 were still related to asthma and rhinitis 10 years later (in 2011).

## Methods

### Sample

**Fig. 1:**
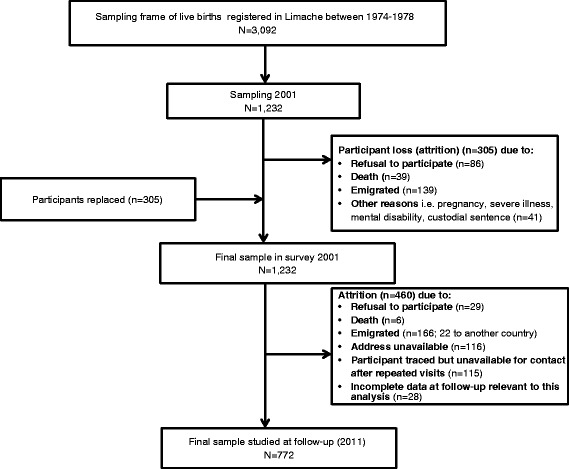
Flow chart of sample selection and attrition in the Limache Cohort (2001 – 2011)

The Limache Cohort study was set up in 2001 with the aim of investigating early and current environmental risk factors for asthma in young adults. This is part of a non-concurrent longitudinal study aimed to assess risk factors in early childhood and in young adults for asthma and poor lung function [10]. A simple random sample of 1,232 subjects was obtained for our initial respiratory disease study based on statistical power estimates using the effects of birth weight differences on lung function as outcome for the respiratory study. Those who could not be included in the study because of death, emigration (11.3 %), serving a custodial sentence, disability or lactation (3.3 %) and unwillingness to participate (7 %) were randomly replaced using the same sampling frame. This decision was taken at protocol stage and was designed to maintain statistical power for hypothesis testing, an important consideration in prospective studies. Ten years later, 796 individuals were traced and enrolled in the follow-up study. The random selection of participants, and attrition at baseline and at follow-up are shown in Fig. 1.

### Outcome measures

We measured lung function (including bronchial hyper-responsiveness [BHR]), respiratory symptoms, and allergy in the 2001 study. Information on respiratory symptoms and related risk factors was also collected. In 2011, participants were contacted again and invited to answer a detailed questionnaire on socio-economic background, lifestyle and respiratory symptoms. In both periods, 2001 and 2011, information on the following asthma symptoms was ascertained using a translated version of the ECRHS questionnaire [11]: “Have you had wheezing or whistling in your chest at any time in the last 12 months?”, “Have you ever had asthma?”, “Has a doctor diagnosed you with asthma? Has a doctor given you advice or treatment for asthma” and “Have woken up at night with shortness of breath in the last 12 months”? The question “Have you had any nasal allergies, including rhinitis in the last 12 months?” was used to estimate nasal allergy and rhinitis. We used the Spanish version of the ECHRS questionnaire that replaced “hay fever” by “rhinitis”, as “hay fever” would not have been understood by most participants in the general population in Spanish speaking countries including Chile.

The tidal breathing method with increasing concentrations of methacholine (0.5, 1.00, 4.00, 8.00 and 16 mg/ml) was used to assess BHR to methacholine challenge at baseline only (in 2001) [12]. BHR was expressed as a continuous measure, the least squares concentration-response slope, analogous to dose-response slope [13]. SPT (measured at baseline only) to the following eight allergens (supplied by Allergy Therapeutics): *D pteronyssinus*, cat fur, dog hair, *Alternaria alternata*, cockroach, a mixture of grass pollens considered the main contributors of pollen in the air in Santiago [14], a mixture of weeds and shrubs, and a mixture of trees. Histamine was used as the positive control and an uncoated Phazet as the negative control. A SPT was positive if the wheal mean diameter was 3 mm or greater. The reference group for allergic status was comprised of those with a negative SPT to all allergens at baseline (*n* = 559). Subjects were considered atopic if they had a positive reaction to any of the allergens tested. Sensitization status was categorized as firstly as ‘sensitization to at least 1 allergen’ (i.e. the participant could have one or more positive sensitization), to give the overall effect of sensitization, and the other three groups to assess whether there was an increasing association between positive reactions to allergens and each of the symptoms studied, as follows: 1) ‘sensitization to one allergen (any)’ (i.e. the participant has a single positive sensitization to any one allergen), 2) sensitization to 2 allergens, and 3) sensitization to 3 or more allergens. In the analysis the reference group were those with no sensitization to any of the eight allergens.

Body weight was recorded using a standard beam balance scale, with subjects standing barefoot and wearing light clothing [15].

### Statistical analyses

Generalized estimating equations (GEE) using equal correlation model were applied to estimate the longitudinal change in the prevalence of symptoms in the population [16] adjusting for gender, age, BMI (continuous), smoking status (current, ex-smoker, never smoker) and atopy (positive to at least one of the eight allergens). GEEs were also used to estimate net changes in prevalence of asthma stratified by the risk factors gender, smoking status, atopy, and response to methacholine challenge and examined unadjusted and adjusted by the variables in the model. The longitudinal association between sensitization status at baseline (2001) and net changes in the prevalence of self-reported respiratory symptoms at follow-up were examined using logistic regression controlling for potential confounders. We added a weighting factor to all analyses to account for the attrition of the cohort between phases 1 and 2 of the cohort study. This factor is based on the reciprocal of the probability of losses, which considers the differences between participants and non-participants [17].

The STATA package version 13.0 was used for statistical analyses.

## Results

The sample distribution of respiratory symptoms reported in 2001 and 2011, and related risk factors are presented in Table 1. There was an attrition rate of 35 % (*n* = 421). 772 individuals had complete data in both surveys. The distribution of smoking was similar to that found in 2001, with a decrease in the proportion of current smokers over the 10 year period (Table 1). The prevalence of reported ‘wheeze in the last 12 months’ decreased 2 % in men and 5 % in women, whilst the prevalence of ‘doctor diagnosed asthma’ increased slightly in both groups. ‘Woken at night with shortness of breath’ increased by 5 % in males and remained stable in women (Table 1).

**Table 1:** Sample distribution of respiratory symptoms and related risk factors in the Limache Cohort in 2001 and 2011

Variables	Survey year 2001 *N* = 1,193		Survey year 2011 *N* = 772	
	Males	Females	Male	Female
	*N* = 544 (45.6 %)	*N* = 649 (54.4 %)	*N* = 282 (36.5 %)	*N* = 490 (63.5 %)
Age (years) mean (SD)	24.9 (1.6)	24.7 (1.6)	35.4 (2.2)	35.1 (2.0)
Smoking status (%) Never	129 (23.7)	253 (39.0)	73 (21.8)	176 (35.8)
Ex-smokers	48 (8.8)	77 (11.9)	22 (26.1)	107 (21.8)
Current	367 (67.5)	319 (49.1)	148 (52.1)	208 (42.4)
Weight (kg) Median (IQR)	70.1 (63.0–77.9)	61.5 (54.9–70.0)	77.9 (69.5–86.5)	69.1 (61.4–78.3)
Adult height (cm) Mean (SD)	168.1 (6.1)	156.4 (5.5)	168.6 (4.9)	156.9 (6.2)
BMI (kg/m^2^) Median (IQR)	24.7 (22.6–27.2)	25.1 (22.6–28.6)	27.3 (24.6–30.1)	28.2 (25.4–31.6)
Wheeze in the last 12 months	26.3	28.4	22.7	23.1
Ever had asthma (%)	3.1	5.9	3.9	7.1
Dr diagnosed asthma (%)	2.6	5.4	3.9	7.1
Woken by shortness of breath in the last 12 months (%)	10.7	16.5	14.2	18.0
Nasal allergies including rhinitis in the last 12 months (%)	10.5	22.0	18.4	30.8
At least 1 asthma symptom and nasal allergies (n, %)^a^	36 (63 %)	82 (58 %)	27 (52 %)	79 (53 %)
Positive skin prick test (SPT) to at least one allergen (data obtained in 2001) (%)	26.7	26.4	28.7	26.9
Positive response to methacholine (data obtained in 2001) (%)	7.9	16.0	7.5	16.9
Educational level achieved				
Primary	126 (23.1)	120 (18.5)	69 (24.5)	106 (21.6)
Secondary	290 (53.3)	348 (53.6)	159 (56.4)	269 (54.9)
Higher	128 (23.5)	181 (27.9)	54 (19.2)	115 (23.5)

^a^Proportion from the total of people reporting having nasal allergies including rhinitis in each group

We investigated the level of bias of the sample in 2011 by comparing the characteristics of participants and non-participants in the follow-up study based on 2001 results, with no differences between participants and non-participants in 2001 (data on Additional file 1: Table S1).

The adjusted net changes of respiratory and allergic symptoms are presented in Table 2. The prevalence of wheeze decreased 3.4 % over ten years, whilst other asthma-related symptoms showed a slight increase, though none of these were statistically significant (Table 2). The net change of nasal allergies was statistically significantly positive (0.8 % per year; 95 % CI 0.47 to 1.18). As current wheeze is the most commonly investigated symptom to ascertain current asthma, we also investigated the pattern of change stratified by potential modifiers (Table 3). Regardless of smoking status, wheeze decreased over 10 years, but the decrease in each group was not significant, although the group of ex-smokers was too small for inference when adjusted for covariates. A reduction in the net change prevalence of current wheeze was observed in those with a negative BHR response, or no atopy, as well as in those who had nasal allergies (Table 3).

**Table 2:** Net changes in prevalence of symptoms of asthma, diagnosed asthma and nasal allergy including rhinitis between 2001 and 2011

Respiratory outcome	*N* (%) in 2001	Net change per year % (95 % CI)^a^
Wheeze in the last 12 months	327 (27.4)	−0.34 (−0.71 to 0.02)
Ever had asthma	55 (4.6)	0.06 (−0.06 to 0.02)^b^
Doctor diagnosed asthma	49 (4.1)	0.15 (−0.004 to 0.30)^c^
Woken by shortness of breath in the last 12 months	165 (13.8)	0.25 (−0.06 to 0.57)
Nasal allergies including rhinitis in the last 12 months	200 (16.8)	0.82 (0.47 to 1.18)^b^

^a^Adjusted for gender, age, BMI, smoking and atopy

^b^BMI not controlled for due to non-convergence when included

^c^BMI, age and smoking not controlled for due to non-convergence when included

**Table 3:** Changes in prevalence of wheeze in the last 12 months 2001–2011 stratified by potential modifiers

Stratification	Category	*N* (%) in 2001	Unadjusted net change % (95 % confidence Interval)	Adjusted net change % (95 % confidence Interval)
Gender^a^	Males	143 (26.3)	−0.16 (−0.71 to 0.39)	−0.27 (−0.81 to 0.27)
	Females	184 (28.4)	−0.59 (−1.08 to −0.10)	−0.48 (−0.96 to −0.004)
Smoking status^b^	Never smoker	39 (17.6)	−0.25 (−0.81 to 0.31)	−0.35 (−0.95 to 0.24)
	Continuous smoker	115 (36.4)	−0.22 (−0.85 to 0.40)	−0.22 (−0.83 to 0.39)
	New smoker (started after 2001)	9 (26.5)	−0.45 (−2.88 to 2.00)	−0.58 (−2.95 to 1.79)
	Ex-smoker (gave up after 2001)	5 (21.7)	−2.00 (−2.91 to −1.02)	convergence not achieved
Atopic status (SPT)^c^	Positive	111 (35.1)	−0.28 (−1.10 to 0.51)	−0.26 (−1.03 to 0.51)
	Negative	216 (24.6)	−0.43 (−0.84 to −0.02)	−0.41 (−0.82 to −0.003)
Methacholine challenge^c^	Positive	57 (38.8)	−0.48 (−1.57 to 0.62)	−0.27 (−1.41 to 0.88)
	Negative	270 (25.8)	−0.37 (−0.76 to 0.01)	−0.39 (−0.76 to −0.01)
Nasal allergies including rhinitis in last 12 months^c^	Positive	98 (49.0)	−1.90 (−2.96 to −0.84)	−1.90 (−3.00 to −0.83)
	Negative	229 (23.1)	−0.10 (−0.49 to 0.28)	−0.10 (−0.46 to 0.28)

^a^Adjusted model included the confounders smoking, SPT, and methacholine challenge

^b^Adjusted model included the confounders gender, SPT, and methacholine challenge

^c^Adjusted model included the confounders gender, and smoking

Sensitization at baseline to either *Dermatophagoides pteronyssinus*, cat fur, cockroach, blend of grass and mixture of weeds or shrubs in 2001 was associated with wheeze in the last 12 months and ever had asthma in 2011 (Table 4). Only a mix of tree allergens was associated with woken by shortness of breath in the last 12 months in 2011. Nasal allergies in 2011 were positively related to most allergens in the unadjusted models in 2001, but it was not any longer significant for cockroach, dog hair and *Alternaria Alternata* in the adjusted model (Table 4). In the analysis assessing generic sensitization, having wheeze in the last 12 months, ever asthma, or nasal allergies in 2011 were related to being sensitized to at least one allergen in 2001 (Table 5). There was no increasing trend of association between the number of sensitizations in 2001 and prevalence of any of the symptoms in 2011, but the association was strongest for ever-asthma in those sensitized to three or more allergens. Waking with breathlessness in 2011 was not associated with sensitization in 2001 (Table 5).

**Table 4:** Risk of asthma symptoms and nasal allergies in 2011 according to allergic status at baseline (2001)^a^

Allergic status in 2001 (positive SPT)	Symptoms reported in 2011 (n)							
	Wheeze in the last 12 months (*n* = 182) OR (95 % CI)		Ever had asthma (*n* = 48) OR (95 % CI)		Woken by shortness of breath in the last 12 months (*n* = 131) OR (95 % CI)		Nasal allergies including rhinitis (*n* = 205) OR (95 % CI)	
	Unadjusted	Adjusted	Unadjusted	Adjusted	Unadjusted	Adjusted	Unadjusted	Adjusted
*Dermatophagoides Pteronyssinus* (*n* = 213)	1.59 (1.05–2.40)	1.31 (0.84–2.05)	3.41 (1.82–6.36)	2.49 (1.06–5.85)	1.38 (0.86–2.21)	1.38 (0.85–2.25)	3.41 (2.31–5.03)	3.56 (2.35–5.40)
Cat fur (*n* = 117)	1.86 (1.12–3.10)	1.76 (1.01–3.05)	3.54 (1.73–7.24)	2.95 (1.15–7.53)	1.53 (0.85–2.75)	1.52 (0.87–2.66)	2.52 (1.54–4.11)	1.88 (1.12–3.13)
Dog hair (*n* = 67)	1.66 (0.86–3.20)	1.47 (0.72–2.98)	3.59 (1.54–8.34)	1.53 (0.79–2.95)	2.06 (1.02–4.16)	1.93 (0.94–3.97)	2.36 (1.27–4.48)	1.73 (0.88–3.38)
Cockroach (*n* = 97)	2.21 (1.26–3.85)	2.09 (1.13–3.86)	1.77 (0.71–4.41)	1.16 (0.39–3.51)	1.41 (0.72–2.77)	1.30 (0.65–2.58)	1.36 (0.77–2.40)	1.31 (0.70–2.43)
*Alternaria alternata* (*n* = 63)	1.13 (0.55–2.34)	1.00 (0.48–2.09)	2.09 (0.76–5.69)	1.67 (0.64–4.35)	1.09 (0.47–2.55)	1.07 (0.47–2.42)	2.31 (1.21–4.36)	1.94 (0.99–3.79)
Blend of grass and pollens^b^ (*n* = 162)	1.96 (1.24–3.08)	1.78 (1.08–2.92)	2.67 (1.34–5.33)	2.94 (1.11–7.79)	1.32 (0.77–2.24)	1.29 (0.75–2.19)	2.44 (1.58–3.77)	2.40 (1.49–3.80)
Mixture of weeds and shrubs^c^ (*n* = 119)	1.67 (0.99–2.82)	1.77 (1.01–3.12)	2.53 (1.20–5.56)	2.38 (0.80–7.12)	1.03 (0.54–1.96)	1.10 (0.59–2.10)	2.01 (1.22–3.31)	1.84 (1.08–3.13)
Tree (*n* = 122)	1.36 (0.81–2.29)	1.08 (0.61–1.92)	2.11 (0.96–4.60)	1.24 (0.46–3.33)	1.91 (1.10–3.30)	1.98 (1.12–3.50)	2.63 (1.64–4.25)	2.40 (1.44–3.99)

^a^Adjusted models included the following potential confounders: by age, gender, smoking status, BMI, years of school, and for the same symptom in 2001

^b^Oats grass, crested dogstail, cocksfoot, rye grass, meadow grass, vernal bent, brome, meadow foxtail, Timothy, meadow fescue, Yorkshire fog

^c^Weeds mixture included Mugwort, fat hen, ouche, nettle, and plantain, and the mixture of trees included birch, beech, oak-common, alder, ash, hazel, poplar, plane, elm, and willow

**Table 5:** Risk of asthma symptoms and nasal allergies in 2011 by combined sensitization status at baseline (2001)

Allergic status in 2001 (positive SPT)	Symptoms reported in 2011 (n) OR 95 % CI^a^							
	Wheeze in the last 12 months (*n* = 182)		Ever had asthma (*n* = 48)		Woken by shortness of breath in the last 12 months (*n* = 131)		Nasal allergies including rhinitis (*n* = 205)	
	Unadjusted	Adjusted	Unadjusted	Adjusted	Unadjusted	Adjusted	Unadjusted	Adjusted
Sensitized to at least 1 allergen (*n* = 316)	1.96 (1.36–2.82)	1.73 (1.17–2.55)	2.84 (1.54–5.24)	3.28 (1.43–7.53)	1.52 (1.00–2.29)	1.47 (0.96–2.25)	3.34 (2.36–4.74)	3.32 (2.30–4.80)
Sensitized to 1 allergen (any)	1.36 (0.87–2.13)	1.27 (0.79–2.05)	1.56 (0.71–3.45)	1.84 (0.70–4.83)	0.92 (0.54–1.57)	0.88 (0.52–1.48)	2.44 (1.60–3.71)	2.56 (1.64–4.02)
Sensitized to 2 allergens	2.29 (1.24–4.21)	2.23 (1.15–4.32)	1.63 (0.53–5.00)	1.83 (0.41–8.22)	1.27 (0.61–2.67)	1.38 (0.64–2.95)	5.01 (2.78–9.06)	5.32 (2.81–10.10)
Sensitized to 3+ allergens	1.62 (0.94–2.79)	1.45 (0.81–2.60)	4.10 (1.88–8.89)	3.74 (1.22–11.46)	1.45 (0.79–2.64)	1.55 (0.85–2.83)	3.52 (2.11–5.87)	3.67 (2.08–6.47)

^a^Adjusted by age, gender, smoking status, BMI, years of school, and for the same symptom in 2001

Those who were negative to the eight allergens formed the reference group

## Discussion

In this prospective study of young adults, we found an eight per cent net increase over a ten-year period in self-reported rhinitis and nasal allergies, a borderline significant decrease in the prevalence of wheeze in the last 12 months, and an increase of Dr diagnosed asthma and waking up with shortness of breath in the last 12 months. Sensitization to cat fur, cockroach, blend of grass and pollens, or mixture of weeds or shrubs at baseline was associated with wheeze in the last 12 months ten years later. Baseline sensitisation to *Dermatophagoides pteronyssinus*, cat fur, and blend of grass was associated with ever asthma in 2011. Most allergens at baseline were also associated with having nasal allergies in 2011.

Our finding of an eight per cent increase of rhinitis and nasal allergy in 10 years was consistent to the multi-centric ECRHS study finding of a net increase of 7 % over a 10 years follow-up [9]. Both studies demonstrated a net increase in Dr. diagnosed asthma of over 1.4 % in the same period. Similarly, both studies demonstrated no significant changes in wheeze and other self-reported symptoms, although there was a borderline significant trend towards a decrease of wheeze in our study. A possible explanation for the stability of symptoms over time might be related to an improvement in the diagnosis and management of asthma in recent years. However, this is an unlikely explanation in our study, as Limache and Olmue have limited medical resources and given the socio economic status of the participants in our study, not many participants could afford treatment elsewhere. In our assessment of possible modifiers we found that ex-smokers, non-sensitized participants and those with a positive nasal allergy were significantly associated to a decrease in wheeze. A decrease in wheeze is plausible in ex-smokers, but this group is too small to influence the trend in the total sample.

Allergic rhinitis is increasing worldwide [18] and represents a major economic burden for the healthcare system. A nine per cent increase in these young adults is of public health relevance as a high prevalence of allergic rhinitis is associated with significant occupational absenteeism. In our study, allergic rhinitis increased in both sexes, possibly because of an increasing temporal trend of the disease [19]. Asthma increased in women, as expected according to population ageing.

Prospective changes in asthma in population based studies have usually been described as incidence and remission [20–23], with reports of the latter ranging between 5 and 40 % in adults, or up to 70 % in children [21], depending on the period of follow-up and the definition of remission used [24]. In a postal survey, Ekerljung and colleagues found that the 10-year remission of wheeze in the last 12 months was 14.6 % in subjects with asthma aged 20–69 years [21] whilst Bronnimann and colleagues reported remission rates ranging from 70 % in children to 10 % in young adults after 10 years of follow-up [22]. Contrasting trends in nasal allergies (increasing) and asthma (stable) have also been observed in Northern Italy with repeated cross-sectional surveys [25]. Our study adds to knowledge by demonstrating that the increase of nasal allergies is occurring not only between individuals over time, but also within individuals. We decided not to estimate incidence because a lack of symptoms of asthma at baseline does not preclude the possibility that a participant may have previously had such a symptom and also because of the possibility of information bias in the responses at each survey. Such eventualities would have provided inaccurate incidence estimates. The small net increase of 1.4 % in the proportion of adults answering ‘yes’ to the doctor diagnosed asthma question might indicate a slight increase in the diagnosis of asthma in Limache over a 10 year period. Such small increase might also be a reflection of better diagnostic skills of asthma in the area. Based on the Tucson Epidemiologic Study [26] we would have expected rhinitis to be an independent risk factor for onset asthma and to influence the trajectory of wheeze over 10 years. However, this was not the case in this adult population.

Symptoms of asthma severity were very infrequent and therefore excluded from our analyses. This may happen because of the limited health care resources available in a semi-rural area. This was one of the reasons why we focused analysis in the question ‘waking up at night with shortness of breath’. This symptom has been used with other questions to estimate asthma severity [27], but we found no evidence of association between waking with shortness of breath and between wheeze, and sensitization at baseline, in contrast to rhinitis or nasal allergy in 2011. This finding highlights the need to investigate the accuracy of the responses to the question waking up at night with shortness of breath in the general population, especially in rural and semi-rural areas in intermediate development countries. We found no association between having a positive BHR at baseline and changes in asthma symptoms at follow-up, which might suggests that changes in asthma prevalence could be related to other environmental exposures in this population.

Atopy has been linked to the development of asthma in children and adults [28]. We found a higher risk of having wheeze in the last 12 months and that of ever having asthma in young adults who were sensitized to *Dermatophagoides Pteronyssinus*, cat, cockroach, pollen, or trees in 2001. These associations remained statistically significant when using 2011 data after adjusting for sensitization status at baseline, which suggests that allergens might contribute to persistence of asthma symptoms. Sensitization played a more important role for the symptoms “nasal allergies including rhinitis” in the follow up study, as reflected by the high odds ratios. Contrary to another epidemiological study [29] we did not observe that being sensitized to multiple allergens was associated with higher risk of current wheeze.

Specific sensitization in the etiology and persistence of asthma has been shown in relation to several allergens. *Dermatophagoides Pteronyssinus,* one of the most commonly distributed indoor allergens, has been proposed as having the strongest causal association with asthma symptoms in adults [30]. A small longitudinal study looking at BHR showed that after a 4 year period sensitization to house dust mite was a risk factor for more broncho-reactivity to BHR, whilst exposure to cat fur was not associated to measures of asthma, including BHR [29]. In our study cat fur but not dog hair was found to be associated with asthma and rhinitis symptoms. Cat dust is known to be a potent allergen with a high concentration of Fel d1 and d4 [31, 32], it is also lighter in weight than dog’s dust, which means it stays in the environment for a longer period, which might partly explain our findings. Furred pet allergens are also passively transferred from one environment to another [32]. The combination of widespread exposure to cat allergens and high prevalence of sensitization to cat suggests that a substantial proportion of individuals with asthma are at risk for cat allergen-induced asthma symptoms. In fact, several studies have directly linked animal allergen exposure to poorer asthma outcomes among animal-sensitized patients with asthma [33].

The strengths of our study were the use of a comprehensive range of allergens and the BHR assessment at baseline, the use of an internationally validated and standardized questionnaire to assess symptoms of asthma and related risk factors and a satisfactory response rate of 65 % over a 10-year period. We also added a weighting factor to allow for the losses observed at follow-up. The area where the study took place is mainly devoted to agriculture and tourism, with a low industrial infra-structure, low road traffic, far from big cities and the Andes (i.e. not locked inside mountains) that allows for a lower accumulation of air pollutants than that observed in the capital, Santiago [34]. These factors make us suggest that the air pollution is not a major problem, in spite of the agricultural and touristic growth of the area. Measurement error is inherent to longitudinal studies, and can lead to biases in the estimates of asthma rates. We attempted to reduce this risk of bias by reporting net changes.

A caveat of our study is that we based the definition of asthma on self-reported symptoms only. Although these are relatively reliable as a proxy for asthma in epidemiological studies, data on BHR at follow-up was not available. The sample size of the study was not sufficiently large for subgroup analysis of those that stop smoking between 2001 and 2011.

## Conclusion

In a cohort study of young adults, symptoms of asthma remained stable over a 10-year period. Our results suggest that sensitization to common allergens plays a role in the reporting of asthma symptoms and rhinitis 10 years later, but are not good predictors of asthma symptoms. This longitudinal study has demonstrated a marked increase in nasal allergy and rhinitis within individuals in contrast with a stable or small decrease of wheeze over a 10 years period. The underlying causes for these contrasting trends are worth studying.

## Abbreviations

BHR, bronchial hyper-responsiveness; ECRHS, European Community Respiratory Health Survey; GEE, generalized equation estimates; SPT, skin prick test

## Additional file

Additional file 1: Table S1. Comparison of baseline characteristics of respondents and non-respondents (measured in 2001). Description of characteristics of respondents and non-respondents participating in the Limache Cohort Study, as measured at baseline (2001). (DOCX 17 kb)
