# Supplementary material for: Changes in symptoms of asthma and rhinitis by sensitization status over ten years in a cohort of young Chilean adults
Source: BMC Pulm Med. 2016 Aug 8;16:116. doi: 10.1186/s12890-016-0273-6 (PMC4977698; doi:10.1186/s12890-016-0273-6)
Supplement: Additional file 1: Table S1. — Comparison of baseline characteristics of respondents and non-respondents (measured in 2001). Description of characteristics of respondents and non-respondents participating in the Limache Cohort Study, as measured at baseline (2001). (DOCX 17 kb) [file 12890_2016_273_MOESM1_ESM.docx]

**Table A1 Comparison of baseline characteristics of respondents and non-respondents (measured in 2001)**

| Variables | Respondents in 2011  N= 1,193 | | Non-respondents survey year 2011  Males and females | |
| --- | --- | --- | --- | --- |
|  | Male  N= 282 | Female  N= 490 | Males  (261) | Females  (n=159) |
| Age (years) mean (SD) | 24.9 (1.6) | 24.7 (1.6) | 24.9 (1.7) | 24.6 (1.5) |
| Smoking status (%) Never  Ex-smokers  Current | 129 (23.7) | 253 (39.0) | 48 (18.3) | 56 (35.2) |
|  | 48 (8.8) | 77 (11.9) | 27 (10.3) | 20 (12.6) |
|  | 367 (67.5) | 319 (49.1) | 187 (71.4) | 83 (52.2) |
| Weight (kg) Median (IQR) | 70.1 (63.0-77.9) | 61.5 (54.9-70.0) | 70.1 (63.7 – 78.5) | 60.7 (55 – 70.5) |
| Adult height (cm) Mean (SD) | 168.1 (6.1) | 156.4 (5.5) | 168.1 (5.9) | 157.4 (5.4) |
| BMI (kg/m^2^) Median (IQR) | 24.7 (22.6-27.2) | 25.1 (22.6-28.6) | 25.0 (22.6 – 27.4) | 24.6 (22.3 – 28.1) |
| Wheeze in the last 12 months (%) | 26.3 | 28.4 | 27.9 | 28.3 |
| Ever had asthma (%) | 3.1 | 5.9 | 3.8 | 5.7 |
| Dr diagnosed asthma (%) | 2.6 | 5.4 | 3.1 | 5.7 |
| Woken by shortness of breath in the last 12 months (%) | 10.7 | 16.5 | 11.1 | 17.6 |
| Nasal allergies including rhinitis in the last 12 months (%) | 10.5 | 22.0 | 11.8 | 25.8 |
| Positive skin prick test (SPT) to at least one allergen (data obtained in 2001) (%) | 26.7 | 26.4 | 24.4 | 24.5 |
| Positive response to methacholine (data obtained in 2001) (%) | 7.9 | 16.0 | 8.4 | 13.2 |
| Educational level achieved, N (%)  Primary  Secondary  Higher | 126 (23.1)  290 (53.3)  128 (23.5) | 120 (18.5)  348 (53.6)  181 (27.9) | 57 (21.8)  131 (50.0)  74 (28.2) | 14 (8.8)  79 (49.7)  66 (41.5) |
